# Superglue self-insertion into the male urethra – A rare case report

**DOI:** 10.22088/cjim.11.3.333

**Published:** 2020-05

**Authors:** Mohsen Khalili Fomeshi, Forough Ebrahimtabar, Mir Mohammad Reza Aghagani, Parvaneh Mirabi, Mohammad Darzi

**Affiliations:** 1 Student Research Committee, Babol University of Medical Sciences, Babol, Mazandaran, Iran.; 2 Infertility and Reproductive Health Research Center, Health Research Institute, Babol University of Medical Sciences, Babol, Iran.

**Keywords:** Foreign body, Superglue, Urethra, Self-insertion

## Abstract

**Background:**

Foreign body insertion in the urethra and bladder are relatively rare. These patients usually insert foreign body in urethra for eroticism, inquisitiveness, or as a consequence of disinhibited or disturbed behavior.

**Case presentation:**

Herein, we report a case of 41-year-old man presented with weak stream and suprapubic pain. Due to incontinence, he instilled superglue into his urethra. On perineal examination, a foreign body was palpable in the penile urethra. A 10 cm dried superglue block got out with incision at glance.

**Conclusion:**

Urethral foreign bodies are mostly found on physical examination and clinical history. Although imaging modalities are commonly used for FBs detection, the necessity of imaging modalities are still a controversy.

Urethra and bladder are not the common sites for foreign body ( FBs) insertion (1). Self-insertion is known as the most common cause followed by iatrogenic events and adjacent site migration (2). Bladder FBs are more probably found in female due to hers short urethra (3), however urethral FBs are 1.7 more likely in male (4). The objects that were reported include wires, pencils, pens, fishhooks, metal rods, needles, thermometers, intrauterine contraceptive devices, tampons, and fluids (glue, hot wax) (5). Most of the self-inserted FBs are related to eroticism, inquisitiveness (particularly in children), disinhibited or disturbed behavior (e.g***. ***psychiatric or senile states) and under the influence of drugs particularly alcohol (6). Here, we reported an unusual case of 41-year-old man with dried superglue in urethra. 

## Case presentation

A 41-year-old married male patient presented to our outpatient clinic with complaint of severe dysuria, weak stream and suprapubic pain that had lasted for 1 week accompanied by complete urinary retention and a month history of incontinence. Due to incontinence, he began self-treatment by instilling superglue into his urethra for 2 weeks. The patient had no urination problem during the early days of insertion. After 4 days, he complained about oliguria and dysuria which emerged simultaneously. By the time of referral to our center (2 weeks later), he was suffering from urinary retention. There was no history of psychiatric disorders. On perineal examination, his penis had erected form. A FB was palpable within the penile urethra and brittle fragments of superglue were seen on the meatus. An abdominal ultrasound revealed bilateral mild hydronephrosis, with normal cortical thickness, and without any sign of stone in the bladder. Laboratory examinations were normal. The patient was taken to the operating room urgently. He underwent a cystoscopy however, it failed due to obstruction.

**Figure 1:**
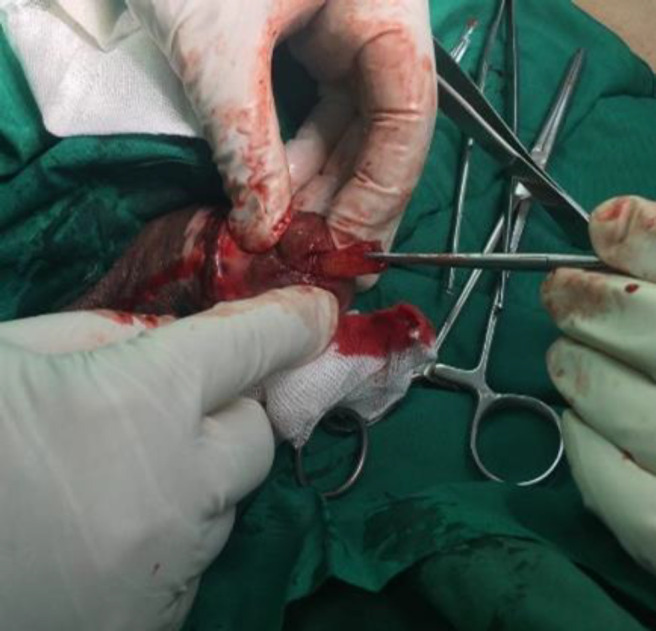
Foreign body removing with glance incision

So the glance was incised in 2 cm length at 6 o'clock position (figure 1). 

**Figure 2:**
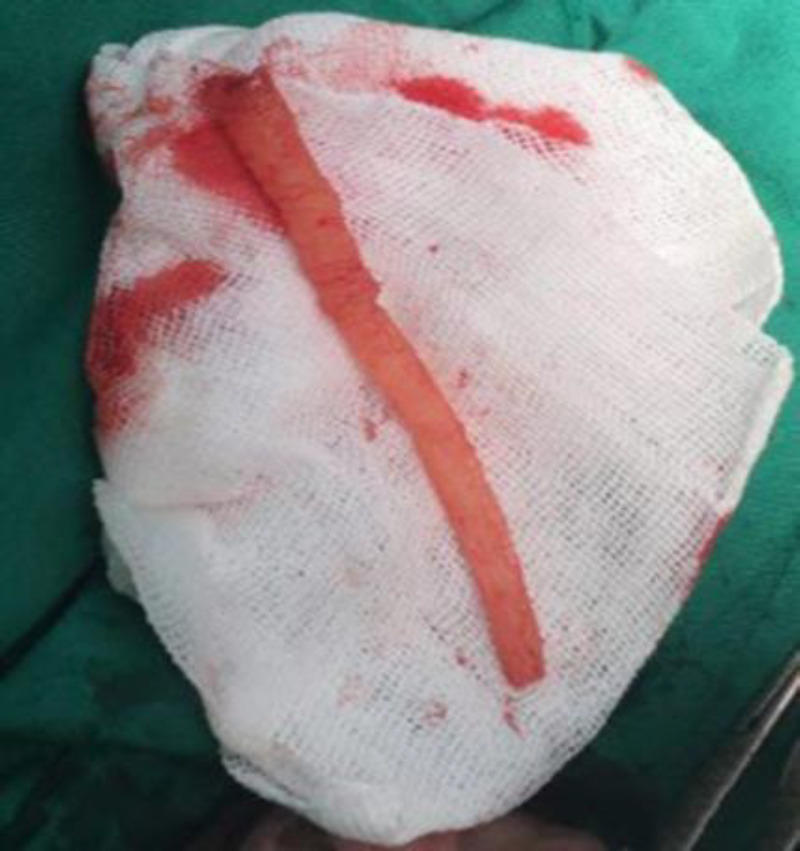
Extracted foreign body (solid block of dried superglue)

The urethra was then washed with 3 liters of normal saline for approximately 15 minutes. During that period we managed to make the solution permeate across the solid material by performing certain maneuvers thereby shedding the adherent layer off the urethral wall. Eventually, a 10 cm rod-shaped solid block of dried superglue (figure 2) came out with the aid of forceps and was removed successfully. The incision was sutured with nylon. After surgery, urination was normal in caliber and direction and the patient was satisfied with urinary drainage. The urine analysis was normal and we did not have requirement for cystoscopy. A 16F Foley catheter was left in place for 10 days. Amikacin 500 mg BD and cefazolin 1gr QID were administrated and he was discharged on the 5th postoperative day without any complication. By the 6^th^ week postoperative, he had no clinical complaints and his uroflow test remained normal.

## Discussion

In our case, the superglue used was an unusual one. This is the 4th case of superglue in the urethra that was reported in the literatures (7-9). Urethral FBs causes are usually associated with self-insertion, migration from adjacent organs or iatrogenic events (3). The most common cause of self-inserted FBs into the male urethra is autoerotic and sexual gratification, especially during masturbation. According to the literatures, in addition to sexual reasons, psychiatric disorders (10), mental confusion (11) and drug intoxication (10) may have great potential to cause self-inflicted FBs. Some patients insert urethral FBs to get relief from urinary symptoms especially in patients of stricture urethra (12) or to commit suicide (12). It is also notable that curiosity particularly among the adolescents can be counted to be an important cause of FB urethral insertion (12). 

Psychiatric evaluation of patients with FBs in the urethra or other orifices revealed no psychiatric problems in some cases and the results were almost controversial. Therefore, there is no consensus on which cases should be evaluated for psychiatric problems (5, 13). According to some literature, psychiatric consultation should be individualized and it would be appropriate in cases with psychiatric problems or FBs insertion history and cases with unusual FBs (13). On the other hand, other literatures indicate the necessity of an initial evaluation for identifying and treating of patients with an underlying mental disorder and also for the prevention of other episodes (5, 10).

Considering females’ short urethra, the FBs can easily slip into the bladder, however, in males the FBs can remain in the urethra for a long time without any symptom or with minimal discomfort (14). Patients usually present with hematuria, dysuria, urinary frequency, strangury, urinary retention, pelvic pain, infections, voiding dysfunction, penile swelling and edema, fever, dyspareunia and leukocyturia (2, 4). 

Physical examination and getting clinical history have been known as diagnostic methods to distinguish FBs in the urethra. The FBs placed at the distal side of the urogenital diaphragm are more palpable than the proximal ones(15). Most of the literatures suggested that pelvic radiography or computed tomography (CT) scan are valuable modalities for evaluating the size, number and structure of FBs and other injuries (3, 4). Some studies demonstrated that small, palpable, and distal located objects are not indicated for radiographic evaluation. Cystourethroscopy is also recommended for the cases that the presence or location of a FB is not well defined. According to this study, pelvic plain film is sufficient to assess radio-opaque objects and if the plain film is insufficient or the object is not radio-opaque, CT scan and ultrasound can be the best alternatives (16).

Most of the FBs located at the distal part of urethra can be successfully removed by endoscopic techniques (i.e. rigid and flexible cystoscopy, grasping forceps, snares and retrieval baskets) (15). In cases with endoscopic treatment failure or in cases of which endoscopic procedure is not an appropriate method, surgical treatment would be a better choice (5). Some case reports recommended that push mobile FBs back into the bladder which manage the patient more easily as compared to endoscopy or surgery methods. Generally, choosing the method depends on size, location, mobility, material and the experience of the urologist with regard FBs. There are other various methods including meatotomy, cystoscopy, internal or external urethrotomy, suprapubic cystotomy, Fogarty catheterization and injection of solvents (15, 17). Aliabad et al’s. study indicate that 6 out of 16 patients with the complaint of urethral FBs were removed successfully via endoscopic techniques. It is worth mentioning that the FBs of these patients were located at the anterior urethra, while the remaining patients with posterior urethra and bladder, FBs underwent open surgery (18).

Accidental superglue application has been reported for different body cavities like ear, nose and urethra that most of them were removed with solvents like acetone. There are 3 case reports with the application of superglue into the urethra, out of which, the FBs were extracted via a surgical management in 2 cases and another one was easily extracted endoscopically (7-9). 

There are some concerns about the use of solvents and their interaction with the urethral mucosa that may damage the urethral mucosa. Acetone, one of the solvents, in the animal study has been used for inducing mucosal damage resulting in a decrement of bladder capacity (8). In the present case, the patient instilled superglue in his urethra for two weeks that caused weak stream and incomplete emptying which finally led to complete urinary retention in the last day. The endoscopic removal failed because of adhesion of the superglue to the urethral wall, so surgery was the best choice. As a conclusion, urethral FB can be diagnosed without any imaging modalities if patient confesses for FB insertion or FB is distinguishable with physical examination and palpation. However, after removing the FB, imaging or cystourethroscopy may be required to check the remnant. 

Self-inflicted FB in the urethra can be fluid like superglue that its’ adhesion to the urethra may make the management difficult. The use of solvents may facilitate superglue removal from the urethra, however, due to the disruptive effect of the solvents on the urethral mucosa, they should not be the first choice for management.
